# Selective decline in the prevalence of slowly adapting type I mechanoreceptors during development

**DOI:** 10.1016/j.ijdevneu.2018.04.001

**Published:** 2018-08

**Authors:** Peter M.B. Cahusac, Solomon Senok

**Affiliations:** a Department of Psychology, Stirling University, Stirling FK9 4LA, Scotland, UK; b College of Medicine, Alfaisal University, PO Box 50927, Riyadh 11533, Saudi Arabia; c Department of Comparative Medicine, King Faisal Specialist Hospital & Research Centre, Riyadh, Saudi Arabia; d Ajman University School of Medicine, PO Box 346, Ajman, UAE

**Keywords:** Merkel nerve endings, Merkel cells, Mechanoreceptors, Slowly adapting mechanoreceptors, Developmental changes, Neuroplasticity

## Abstract

Merkel nerve endings are identified physiologically as slowly adapting type I mechanoreceptor units. They are important for fine acuity tactile perception. We examined the effect of age on the electrophysiological availability of different types of slowly adapting mechanoreceptor units. Using 6–50 week old rats, we observed an obvious decline with age in the probability of recording from St I units of the deep vibrissal nerve. The precipitous decline occurred between ages 6–14 weeks and then stabilized. By contrast, the prevalence of St II units, the other type of slowly adapting mechanoreceptor, remained constant over the age range studied. These observations correlate with anatomical findings reported elsewhere.

## Highlights

• Electrophysiological recordings were made from sinus hair follicles in vitro from rats aged from 6 to 50 weeks old.
• The prevalence of slowly adapting type I mechanoreceptors declined abruptly from 6 to 14 weeks, stabilizing thereafter.
• In contrast, the prevalence of slowly adapting type II mechanoreceptors did not show any decline over the study period.
• These findings suggest that there is pruning of Merkel nerve ending mechanoreceptors with age.
• The importance and triggers for these changes remain to be established.

## 1 Introduction

Understanding changes in sensory systems during development and aging is important to facilitate therapeutic intervention, adaptation and compensation (Verdú et al., 2000). Studies have shown that vibrotactile sensation declines with age (Deshpande et al., 2008) which may partly explain the greater susceptibility of the elderly to falls. In contrast, the sensory nerve fibres involved in pain perception show no such decline (Jimenez-Andrade et al., 2012). Slowly adapting type I (SA I) mechanoreceptors in primates, including humans, are responsible for high resolution pattern discrimination by the fingertips (Johnson, 2001). Terminations of SA I afferents arborize in the epidermis to give rise to disc-like endings apposed to Merkel cells and are known as Merkel nerve endings. The highest concentration of Merkel nerve endings in rodents is found in their sinus hair follicles (Halata et al., 2003). Many animals bear prominent sinus hairs which act as complex tactile organs to sense their immediate environment, especially useful in nocturnal species. Their use aids spatial navigation, hunting and foraging. The rat’s vibrissa system has a discriminative ability similar to that of primate fingertip tactile perception (Carvell and Simons, 1990), and this system is widely used as a model for high acuity tactile sensation. Rat SA I mechanoreceptors, known as St I mechanoreceptors, are located in the sinus hair follicle complex and play an important role in the well-developed discriminative abilities of rats and in other whisker-bearing animals. The anatomical identity of electrophysiologically recorded St I units has been conclusively confirmed as Merkel nerve endings (Ikeda et al., 2014; Maksimovic et al., 2014). An age-related decrease in neurotrophin expression, particularly neurotrophin-3 and neurotrophin-4, has been correlated with a reduction in number and density of Merkel nerve endings and associated large diameter fibres in rat sinus hair follicles (Bergman et al., 2000). Other anatomical work has shown that Merkel endings decline most from 6 to 120 weeks of age in rats (Fundin et al., 1997). The present study analysed the decline in Merkel nerve endings from an electrophysiological perspective, comparing the prevalence of St I mechanoreceptors with St II mechanoreceptors over time. This work has previously appeared in abstract form (Cahusac and Senok, 2011).

## 2 Methods

The Ethics Committee of Stirling University Department of Psychology approved the use of animals in this study. All procedures complied with UK Home Office and European Directive 86/609/EEC.

Data from a total of 112 male albino Wistar-derived rats were included in analyses. Animals were aged between 6 and 50 weeks old, were fed ad libitum in conventional cages. Animals were used as made available by the animal facility, and their ages can be considered as semi-random between the limits of 6 and 50 weeks old. Animals were generally caged with one or two other siblings.

**Fig. 1:**
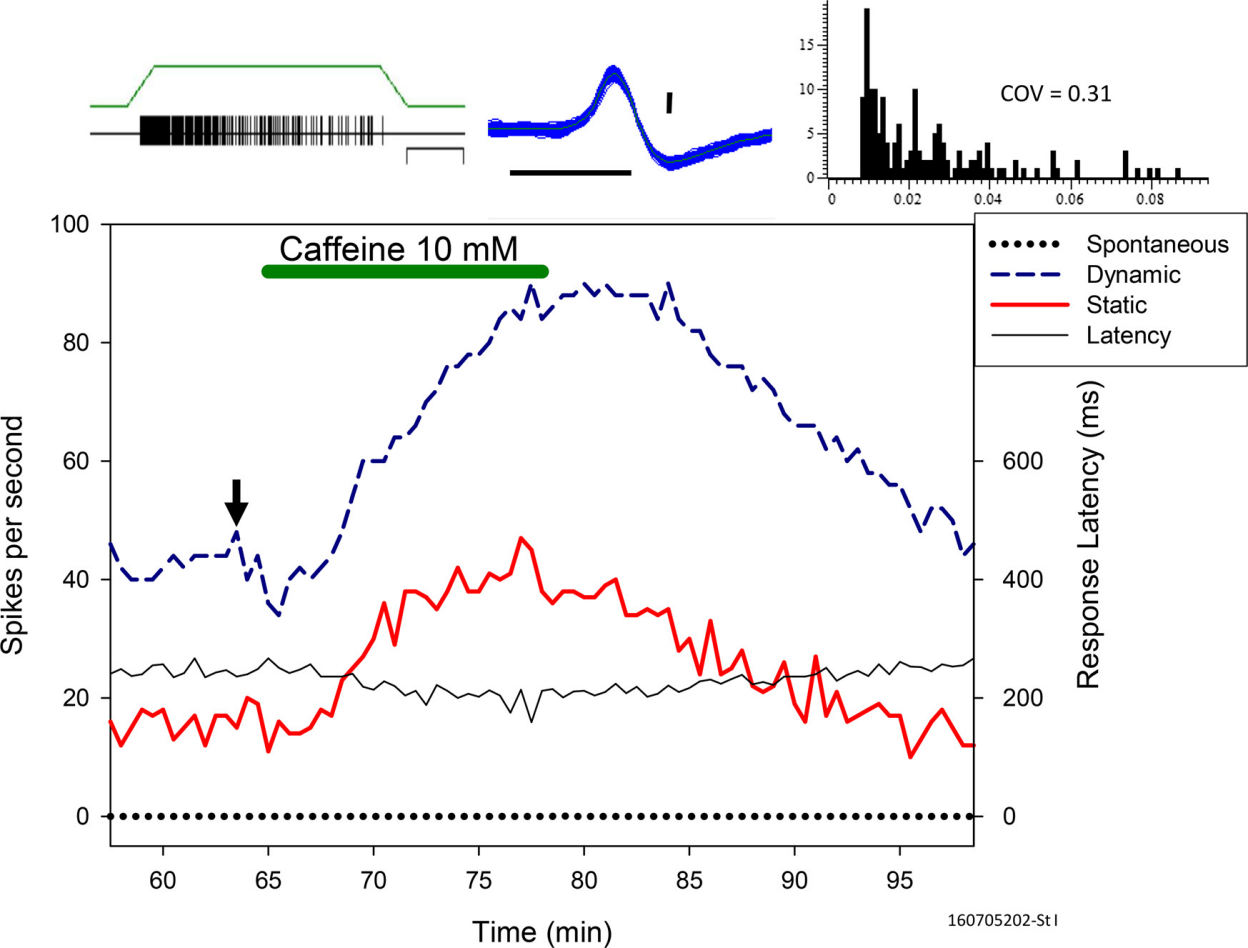
The response enhancing effect of 10 mM caffeine on the firing of a single St I unit from a 33 week old animal. The responses to 1 mm ramp deflection of the sinus hair were increased by the caffeine and the dynamic and static components are illustrated here. The spontaneous firing showed no increase. The caffeine caused an almost immediate decrease in the onset latency of the response, dropping from a previous mean of 248 ms to about 200 ms. After the end of the caffeine application, recovery of all these effects occurred within 20 min. Insets above the graph show the neural activity of a selected window chosen from a typical response indicated by the vertical arrow on the graph. From right to left: the stimulus ramp and recorded spikes (calibration bar 1 s), all 163 overlaid spikes (horizontal bar 1 ms, vertical bar 20 μV), a frequency histogram of interspike intervals (horizontal scale in s), with COV of 0.31.

As previously described (Cahusac and Noyce, 2007), each animal was deeply anesthetised with intraperitoneal urethane (2 g/kg) and then euthanized by urethane injection into the heart. Whisker pads were removed, and the larger sinus hairs with follicles and nerve attached were microdissected out and placed in carbogenated (95% oxygen, 5% carbon dioxide) synthetic interstitial fluid (SIF) (Bretag, 1969) containing NaCl (107.80 mM), sucrose (7.60 mM), NaHCO_3_ (26.19 mM), d-gluconic acid (9.63 mM), d -glucose (5.55 mM), KCl (3.49 mM), NaH_2_PO_4_·2H_2_O (1.88 mM), MgSO_4_·7H2O (0.69 mM) and CaCl_2_ dihydrate (1.90 mM). As described in detail (Baumann et al., 1996), each individual follicle was slit lengthways and desanguinated. The follicle was then pinned onto a Sylgard (Dow Corning, Michigan, USA) rubber platform within a custom-made tissue bath provided by courtesy of Professor K. Baumann, Hamburg. The vibrissa hair shaft was cut down from approximately 5 cm to 5 mm long. A length of 10 mm of the deep vibrissal nerve was retained. The outer sheath of the nerve was stripped off and the nerve bundle divided repeatedly until fine nerve strands could be teased out and attached to a silver recording wire. The recording wire was immersed in Fluorinert (FC-40 Sigma) at the bottom of the bath. Carbogenated SIF perfused the preparation above the Fluorinert (FC-40; Sigma, Gillingham, Kent, UK). Bath temperatures were maintained between 29–33 °C. Electrical activity of nerve fibres was recorded using Digitimer Neurolog equipment (Welwyn Garden City, Herts, UK), amplified, filtered and monitored on oscilloscopes and a loudspeaker. The responsiveness of individual units was assessed by deflecting the vibrissa shaft with fine forceps. Reproducible responses could be obtained by placing the end of the vibrissa inside a 90 cm long glass capillary (1.2 mm outer diameter) fixed to a Piezoelectric trimorph bending actuator (35 mm × 6 mm × 0.6 mm; Morgan Matroc Vernitron Piezoelectric Division, Bedford, Ohio, USA). The actuator was driven by 10–200 V to provide deflections of the vibrissa of up to 2 mm, sufficient to elicit approximately 75% of the unit’s maximal response firing. Different types of units could be identified by their characteristic response properties. Type I (St I) units exhibited irregular firing with a large coefficient of variation (COV) > 0.1. Type II (St II) units exhibited highly regular firing with a COV < 0.1. The differences were clear from online inspection of plots of interspike intervals against their serial position in time (Iggo and Muir, 1969). Additional confirmation could be obtained by the discriminating effects of 10 mM caffeine (Sigma, Gillingham, Kent, UK) (Senok and Baumann, 1997) and 3 mM tetraethylammonium (TEA, synthesized by Dr. E. Porter, Stirling University, UK) (Senok et al., 2001). An example of the effects of caffeine on a St I unit is shown in Fig. 1. SPIKE2 (Cambridge Electronic Design, Cambridge, UK) computer script controlled stimuli and collected data. Discriminated action potentials were saved as event times and spike shapes via a 1401plus (Cambridge Electronic Design, Cambridge, UK) electronic interface to a computer. All well isolated units with at least 2:1 signal to noise ratio were noted in the lab book during each experiment, regardless of which specific types of units (St I or St II) that the investigators were primarily interested in recording from on that particular day. Analogue temperature, stimulus events and other relevant experimental events were also stored. Selective analogue records were made on an 8-channel video recorder (V-Store, Racal, Weybridge, UK). Non-parametric statistical analyses were done using SPSS v21 (IBM Corp, New York, USA). Grouped medians and their associated bootstrap standard errors were calculated for the number of units per experiment for the different ages.

## 3 Results

**Fig. 2:**
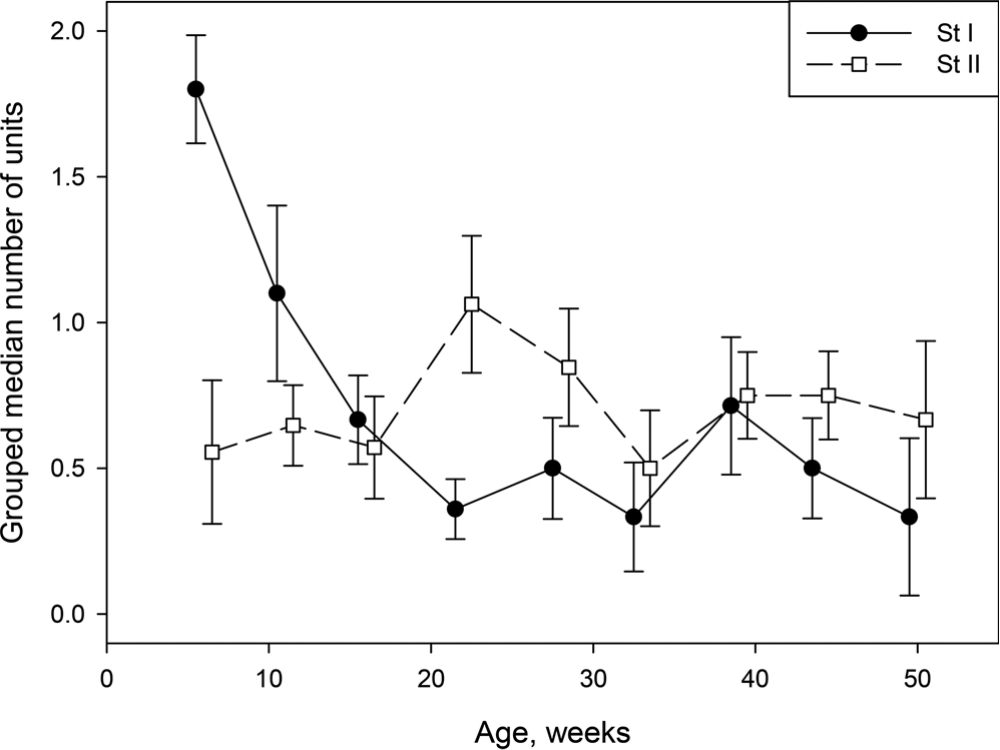
Plot of grouped median number of units against age. The medians for St I and St II units are plotted separately. From 6 to 14 weeks there is a sharp decline in the median number of St I units encountered in experiments. St I data are shown by solid line and filled circle and St II data are shown by dashed line and unfilled square, as indicated by the key. The error bars are ± the associated bootstrap standard error. The points and error bars are jittered by 0.5 week in order to avoid overplotting of points.

On average, almost 2 (median = 1.8) St I units were encountered per animal in experiments performed in the youngest rats aged 6 weeks (see Fig. 2). However this number declined markedly with age, so that in 16 week old animals the average was down to one third of this value with median = 0.67 units. This lower level persisted through the remaining 34 weeks of study with an average median of 0.49 units per experiment (i.e. only one unit would be expected for every 2 experiments run). A non-parametric one-way ANOVA revealed a statistical difference across the different age groups (*χ*^2^(8) = 29.2, *p* < 0.001). Pairwise comparisons across age groups using Mann-Whitney test revealed that the frequency of St I units at 6 weeks was not statistically different from 11 week animals (*p* = .092) but was greater compared to all the older age groups (*p* < 0.001). The frequency of St I units in 11 week animals was also different from older age groups (*p* = 0.01).

By comparison, the occurrence of St II units did not show statistical changes with age, and was relatively constant over the study time period. In the youngest rats there was a median of 0.56 units per experiment. They appear to briefly rise to 1.06 units per experiment in 22 week old animals, returning back to an average of 0.70 units per experiment from week 28 onwards. It seems likely that these differences over time represented variability since a non-parametric one-way ANOVA revealed no indication of statistical differences across the different ages (*χ*^2^(8) = 5.61, *p* = 0.691). No pairwise comparisons for St II units were statistically different for 22 week old animals compared with any other weeks (0.123 < *p* < 0.516). In summary, there is a clear effect of age for St I units, which show a rapid decline in numbers from the youngest to the older animals, while there were no obvious age differences for St II units.

## 4 Discussion

This study shows that there is a clear relationship between the age of rat and the frequency of encountering St I units using electrophysiological sampling of single nerve fibres of the deep vibrissal nerve. The frequency of St I units declines dramatically from the earliest studied animals, aged 6 weeks, to older animals up to 50 weeks old.

The decline we observed may have several explanations. First it is possible that as animals age it becomes more difficult to record adequately from single nerve fibres, perhaps due to developmental changes in the nerves and nerve sheaths. Possibly the electrical signal generally declines with age, making it more difficult to clearly identify single nerve recordings in older animals. This explanation seems highly unlikely since the other type of unit studied here, St II unit, did not show the same changes. St II units were recorded with the same frequency across all ages. There may have been bias by the investigators, so that only certain types of units were sought and recorded from and these happened to correlate with the age of the animals used. This is also highly unlikely since in each experiment the investigator noted down all units which had a signal-noise ratio of at least 2:1 and sufficient data collected for conclusive classification (including the COV and results from pharmacological tests). These units were recorded irrespective of which particular types of units were being targeted in a given experiment. The current study addressed the issue of prevalence of different mechanoreceptor types.

It is possible that the decline in St I units that we observed may follow the principle of “use it or lose it”. A recent study has shown that rats exposed to environmental enrichment or deprivation (whisker trimming) had effects on nerve terminals within the trigeminal nuclei (Fernández-Montoya et al., 2018). Specifically, in the enriched condition, there were increases in the number and size of myelinated varicosities in all the trigeminal nuclei (Fernández-Montoya et al., 2018). Perhaps the decline observed in the current study is most apparent in the artificial and relatively sensory deprived conditions seen in animals kept in laboratory cages. Less or no effect might be observed in wild animals, where animals will need to use their receptors for survival. Changes in other parts of the nervous system have been observed following environmental enrichment (Diamond et al., 1964), and so it is possible that this decline may be influenced by the animals’ sensory experience.

Alternatively, it is possible that the changes we observed, between 6 and 50 weeks of age, correspond to normal developmental changes. That is, the pruning of mechanoreceptors represents a natural maturational event (Cunningham, 1982). Hence, in early life there may be an abundance of Merkel nerve endings which would be important while animals are first exploring their tactile world. With age and experience these endings become less important, and their numbers reduced to a lower level.

Work in mice indicates that postnatal growth and maturation of Merkel nerve endings are increased in transgenic animals that overexpress neurotrophin-3 (Krimm et al., 2004). The same study found that the survival of Merkel nerve endings did not depend on neurotrophin-3 but on innervation (Krimm et al., 2004). The decline in prevalence of St I units seen between 6–14 weeks most likely corresponds to the anatomical reduction in Merkel nerve endings observed between 6–120 weeks by other studies (Fundin et al., 1997). We suggest that there may be two components in play: an early rapid decline from young adulthood due to a pruning of St I mechanoreceptors, and a later decline only observed after 1–2.3 years that is associated with senescence.

## Disclosure

We confirm that there are no actual or potential conflicts of interests concerning financial, personal or other relationships with other people or organisations.
